# Caspase-9, caspase-3 and caspase-7 have distinct roles during intrinsic apoptosis

**DOI:** 10.1186/1471-2121-14-32

**Published:** 2013-07-09

**Authors:** Matthew Brentnall, Luis Rodriguez-Menocal, Rebeka Ladron De Guevara, Enrique Cepero, Lawrence H Boise

**Affiliations:** 1 Departments of Hematology and Medical Oncology and Cell Biology, Winship Cancer Institute of Emory University, 1365 Clifton Road NE Bldg:C, Rm:4012, Atlanta, GA 30322, USA; 2 Sheila and David Fuente Graduate Program in Cancer Biology, University of Miami Miller School of Medicine, Miami, FL, USA; 3 Department of Microbiology and Immunology, University of Miami Miller School of Medicine, Miami, FL, USA

**Keywords:** Caspase, Bid, ROS, Intrinsic apoptosis, Mitochondria, Cell detachment

## Abstract

**Background:**

Apoptosis is a form of programmed cell death that is regulated by the Bcl-2 family and caspase family of proteins. The caspase cascade responsible for executing cell death following cytochrome *c* release is well described; however the distinct roles of caspases-9, -3 and -7 during this process are not completely defined.

**Results:**

Here we demonstrate several unique functions for each of these caspases during cell death. Specific inhibition of caspase-9 allows for efficient release of cytochrome *c*, but blocks changes in mitochondrial morphology and ROS production. We show that caspase-9 can cleave Bid into tBid at amino acid 59 and that this cleavage of Bid is required for ROS production following serum withdrawal. We also demonstrate that caspase-3-deficient MEFs are less sensitive to intrinsic cell death stimulation, yet have higher ROS production. In contrast, caspase-7-deficient MEFs are not resistance to intrinsic cell death, but remain attached to the ECM.

**Conclusions:**

Taken together, these data suggest that caspase-9 is required for mitochondrial morphological changes and ROS production by cleaving and activating Bid into tBid. After activation by caspase-9, caspase-3 inhibits ROS production and is required for efficient execution of apoptosis, while effector caspase-7 is required for apoptotic cell detachment.

## Background

Intrinsic apoptosis is a mitochondrion-centered cell death that is mediated by mitochondrial outer membrane permeabilization (MOMP), results in apoptosome formation, activation of caspase-9 and subsequent activation of effector caspases. Growth factor withdrawal and intracellular stress can induce apoptosis through the intrinsic cell death pathway, while extrinsic apoptosis is initiated through transmembrane death receptors. Initiation and execution of these processes are regulated by the BCL-2 and caspase families of proteins [1,2]. Activation of the BCL-2 family members Bax and Bak results in MOMP and the release of pro-apoptotic proteins, including cytochrome *c*, from the inter-membrane space into the cytosol [3-5]. Cytochrome *c* can then bind Apaf-1 forming the apoptosome and activating caspase-9. Once active, caspase-9 can directly cleave and activate caspase-3 and caspase-7 [6,7].

Effector caspases are responsible for initiating the hallmarks of the degradation phase of apoptosis, including DNA fragmentation, cell shrinkage and membrane blebbing [8,9]. Other characteristics of apoptosis include, mitochondrial remodeling, ROS production and cleavage of a variety of proteins, but the role of caspases in these processes is not fully understood [9-12]. We have previously shown that during intrinsic cell death stimulation caspase-9 and effector caspases have sequential and distinct effects on mitochondria. Caspase-9 can prevent accessibility of cytochrome *c* to complex III in the mitochondria, resulting in increased ROS production, but in the presence of effector caspase activity, ROS production is terminated [13]. Taken together, these data suggest a possible feedback loop on the mitochondria after cytochrome *c* release and caspase activation. Previous studies show that caspase-8 can cleave Bid into tBid, which can remodel the mitochondria, but the role of tBid in intrinsic apoptosis has not been determined [10]. Also, previous data have shown that caspase-9 is a highly specific protease that only cleaves a few proteins, where as caspase-3 and caspase-7 contribute to the majority of cleavage that takes place during apoptosis, but the distinct roles of each caspase is not understood [14]. Based on cleavage-specificity profiles for caspase-3 and caspase-7, it was believed that these caspases were essentially redundant in regards to substrate cleavage during apoptosis [15,16]. However, recent data suggests that caspase-3 and caspase-7 must have distinct functions because mice deficient in these caspases have distinct phenotypes and caspase-3 and caspase-7 have differential activity toward synthetic, natural and purified substrates [17,18]. Therefore, more research needs to be conducted looking into the distinct functions of each caspase during intrinsic apoptosis.

To address these issues, we used genetically manipulated cell lines to study the distinct functions of caspase-9, caspase-3 and caspase-7 during intrinsic cell death stimulation. Here, we show that caspase-9 can remodel mitochondria and increase ROS production by cleaving Bid into tBid. Also, caspase-3 can inhibit ROS production and is the effector caspase necessary for efficient cell killing. In contrast, caspase-7 has no significant role in sensitivity to intrinsic cell death, but it is responsible for ROS production and cell detachment. Taken together, these data suggest that caspase-9, caspase-3 and caspase-7 have distinct roles during intrinsic apoptosis.

## Results

### Caspase-9-cleavable Bid is necessary for ROS production during apoptosis

**Figure 1:**
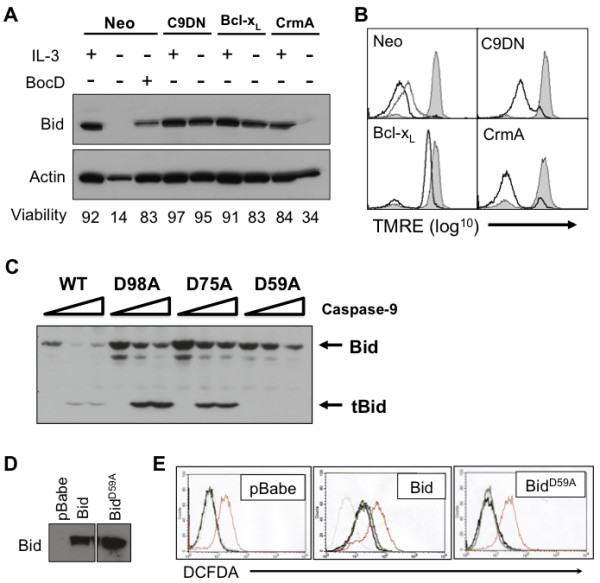
**Bid is cleaved in a caspase-9-dependent fashion during IL-3 withdrawal and is necessary for ROS production. (A**-**B)** FL5.12 Neo (±100 μM BocD-fmk), Bcl-x_L_, Casp9DN and CrmA cells were cultured in the presence or absence of IL-3 for 24 h. **(A)** Bid levels were determined by western blot and viability was determined. **(B)** Loss of membrane potential was determined by flow cytometry (filled histogram (+)IL-3, black line (-)IL-3, grey line (-)IL-3 +BocD). **(C)***In vitro* translated Bid, Bid^D98A^, Bid^D75A^ and Bid^D59A^ were subjected to cleavage by increasing levels of caspase-9 for 90 min. Bid cleavage was determined by western blot. **(D)** Bid and Bid^D59A^ were expressed in Bid^-/-^ MEFs by retroviral transduction and Bid levels were determined by western blot (Bid expression from same blot). **(E)** Bid^-/-^ pBabe, Bid and Bid^D59A^ MEFs were withdrawn from serum for 12 hr. ROS production was determined by flow cytometry. (light grey line (+)FBS, dark grey line (-)FBS, black line (-)FBS +BocD). Treatment with 50 μg/ml antimycin A for 30 min was used as a positive control (red line). Data are representative of at least 3 independent experiments.

FL5.12 cells are a pro-B cell line dependent on IL-3 and can be used as a model for intrinsic apoptosis activation. During IL-3 withdrawal, FL5.12 cells release cytochrome *c* from the mitochondria and the mitochondria change from a normal morphology with numerous narrow cristae surrounded by electron-dense mitochondrial matrix to a remodeled morphology with a large electron-transparent intracristal space. However, introduction of a dominant negative Casp9 inhibits mitochondrial remodeling without affecting the release of cytochrome *c* (Data not shown) [13]. These data suggest that caspase-9 is responsible for mitochondrial remodeling downstream of MOMP during IL-3 withdrawal. It has been shown that Bid can remodel the mitochondria after cleavage by caspase-8 into tBid, therefore we examined if caspase-9 could cleave Bid [10]. We show that during IL-3 withdrawal of FL5.12 cells (Neo), Bid expression is lost and there is depolarization of the mitochondria. However, Bid expression can be rescued by expression of Bcl-x_L_ or Casp9DN, but not CrmA, a caspase-8 inhibitor (Figure 1A and B). Also, blockade with BocD-fmk or Casp9DN causes an incomplete depolarization of the mitochondria, while CrmA has no effect on loss of ΔΨ_m_ (Figure 1B). Since loss of Bid and loss of ΔΨ_m_ were rescued by inhibition of caspase-9 and not inhibition of caspase-8, we examined if caspase-9 could directly cleave Bid. Caspases are known to cleave Bid at three sites, Asp^98^, Asp^75^ and Asp^59^, therefore we determined the sites caspase-9 was cleaving Bid [19]. Bid mutants, Bid^D98A^, Bid^D75A^, Bid^D59A^, were incubated with increasing concentrations of recombinant caspase-9 and while Bid^D98A^ and Bid^D75A^ were cleaved by caspase-9, Bid^D59A^ was not (Figure 1C). These data suggest that caspase-9 can cleave Bid into tBid at aspartic acid 59, which would be consistent with the ability of tBid to function in remodeling the mitochondria during intrinsic cell death.

To further study the affects of caspase-9 cleavage of Bid, we reconstituted Bid^-/-^ MEFs with wild-type Bid, the cleavage mutant Bid^D59A^ or with vector control (pBabe) in order to test the significance of Bid cleavage during intrinsic cell death (Figure 1D). Caspases can regulate ROS production during apoptosis and we have previously shown that in the absence of effector caspase activity, caspase-9 can cause increased ROS production [13]. Therefore, we determined the role of caspase-9 cleavage of Bid on ROS production. Bid^-/-^ pBabe, Bid^-/-^ Bid, and Bid^-/-^ Bid^D59A^ MEFs were subjected to serum withdrawal for 12 hours in the presence or absence of BocD-fmk and ROS production was determined. Bid^-/-^ pBabe MEFs show no increase in ROS production after serum withdrawal, even when effector caspases are inhibited by BocD-fmk, suggesting that ROS production is not initiated by serum withdrawal in the absence of Bid. In contrast, Bid^-/-^ MEFs reconstituted with Bid display an increase in ROS production, which is modestly increased by BocD-fmk treatment, therefore Bid is necessary for ROS production, while effector caspase activity can inhibit ROS production. However, when Bid^-/-^ Bid^D59A^ MEFs are subjected to serum withdrawal there is no increase in ROS production, suggesting that caspase-9 cleavage of Bid is necessary for ROS production during intrinsic apoptosis (Figure 1E).

### Effector caspase-3 and caspase-7 have distinct roles during apoptosis

**Figure 2:**
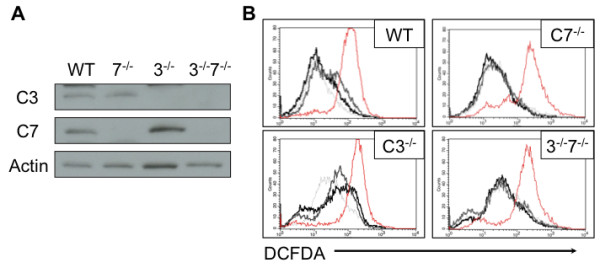
**Caspase-7 and-3 have distinct effects on ROS production during apoptosis. (A)** Expression levels of effector caspases in Casp KO MEFs were determined by western blot. **(B)** WT, Casp7^-/-^, Casp3^-/-^ and Casp3^-/-^7^-/-^ MEFs were withdrawn from serum for 12 hr. ROS production was determined by flow cytometry. Treatment with 50 μg/ml antimycin A for 30 min was used as a positive control. Data are representative of at least 3 independent experiments.

Our previous studies demonstrated that blocking caspases downstream of MOMP resulted in a partial block in loss of ΔΨ_m_[13]. Figure 1B demonstrated that blockade with Casp9DN or BocD-fmk results in an incomplete depolarization of mitochondria, while CrmA has no effect on loss of ΔΨ_m_. This suggests that the initial loss of ΔΨ_m_ is caspase independent and likely reflects the effects of MOMP. Consistent with this possibility, Bcl-x_L_ expression blocks loss of ΔΨ_m_. However, we have shown that incomplete depolarization does not necessarily result in ROS production, as inhibition of caspase-9 does not induce ROS [13]. This suggests that the requirements for ROS production and total loss of ΔΨ_m_ are independent. While caspase-9 cleavage of Bid can account for the generation of ROS, the data suggests that accumulation of ROS is related to downstream inhibition of caspase-3 and/or caspase-7 by BocD-fmk. We have previously shown that BocD-fmk inhibits all DEVDase activity during IL-3 withdrawal-induced death [13]. Therefore, our previous studies could not discriminate between inhibition of caspase-3 and caspase-7. To determine if caspase-3 and caspase-7 play specific roles in the regulation of ROS production during intrinsic cell death, WT, Casp7^-/-^, Casp3^-/-^ and Casp3^-/-^7^-/-^ MEFs were subjected to serum withdrawal for 12 hours and ROS production was determined. Casp KO MEFs did not compensate for the loss of one effector caspase by increasing expression of the other caspase (Figure 2A). WT MEFs display an increase in ROS production after serum withdrawal and consistent with our findings in IL-3 withdrawal and chemotherapy-induced death, this is augmented by the addition of BocD-fmk [13,20]. Interestingly, Casp7^-/-^ MEFs display no increase in ROS production following serum withdrawal and surprisingly BocD-fmk had no effect on ROS production. In contrast, in Casp3^-/-^ MEFs an increase in ROS production during serum withdrawal is observed and this is not altered by addition of BocD-fmk (Figure 2B). Together these data suggest that caspase-3 is responsible for limiting ROS production, however they also suggest that caspase-7 may contribute to ROS production. To directly test this possibility, ROS production was determined following serum withdrawal of caspase-3/caspase-7 DKO MEFs. Consistent with the possibility that caspase-7 plays a role in the production of ROS, no increase in ROS production was observed (Figure 2B).

**Figure 3:**
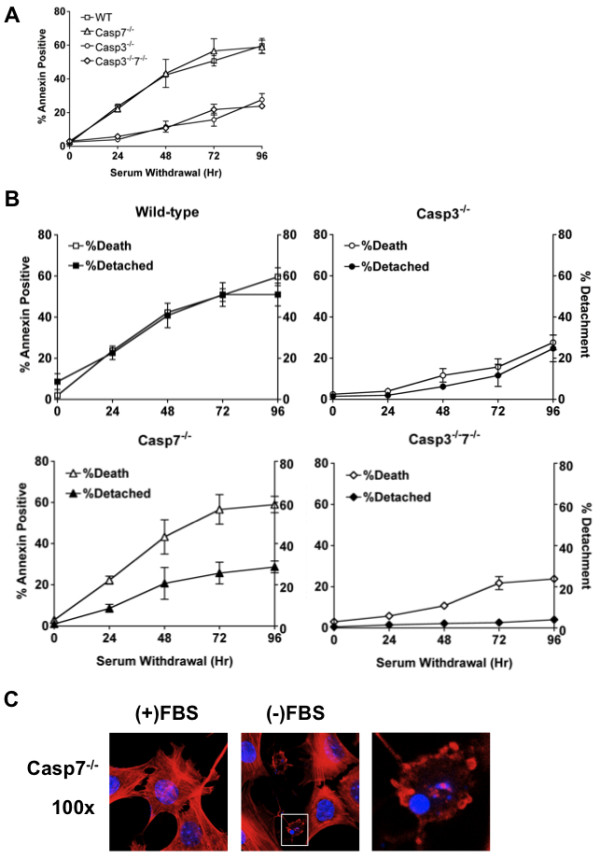
**Caspase-7 and-3 have distinct functions during apoptosis. (A**-**B)** WT, Casp7^-/-^, Casp3^-/-^ and Casp3^-/-^7^-/-^ MEFs were withdrawn from serum for 4 days. **(A)** At the indicated time points, cell death was determined by Annexin V-FITC staining. **(B)** At the indicated time points, percent detachment was determined by separating non-adherent from adherent cells and counting with a hemocytometer. Data are presented as the mean ± SEM of at least 3 independent experiments. **(C)** Casp7^−/−^ MEFs were grown on glass coverslips for 24 hr and then were washed with PBS and the medium was replaced with full medium or serum free medium for 48 hr. After, cells were fixed, stained for actin and DNA and visualized by fluorescent microscopy.

During the course of these studies, we noted differences between cell lines in the amount of cell death and the number of adherent cells remaining following serum withdrawal. Therefore, we wanted to further identify if specific effector caspases were responsible for these phenotypes. A serum withdrawal time course was completed and cell death and percent detachment were determined. While loss of caspase-7 provided no protection from serum withdrawal-induced cell death, caspase-3-deficient cells displayed significant protection. Moreover this protection was not different in DKO cells (Figure 3A). These data suggest that caspase-3 is the dominant executioner caspase and that caspase-7 activation is neither necessary nor sufficient for serum withdrawal-induced cell death. Following serum withdrawal, cell death of WT MEFs correlated with percent of detachment suggesting that all dead cells were detached from the ECM. Casp3^-/-^ MEFs displayed a correlation between cell death and cell detachment following serum withdrawal, while less cells died, the ones that did had detached from the ECM. In contrast, Casp7^-/-^ and Casp3^-/-^7^-/-^ MEFs had significantly lower levels of cell detachment when compared to cell death suggesting that dead cells remained attached to the ECM (Figure 3B). To examine the morphology of dead cells that remained attached to the ECM, we performed a 48-hour serum withdrawal on Casp7^−/−^ MEFs and determined changes in actin organization and DNA by fluorescent microscopy. In complete medium all Casp7^−/−^ MEFs display numerous distinct actin stress fibers and actin rich cell protrusions with a large round nuclei. However, after serum starvation some attached cells display morphological changes consistent with apoptosis, including cell rounding, membrane blebbing, and condensed and fragmented nuclei (Figure 3C). Taken together, these data indicate that caspase-7 is responsible for cell detachment during intrinsic cell death.

**Figure 4:**
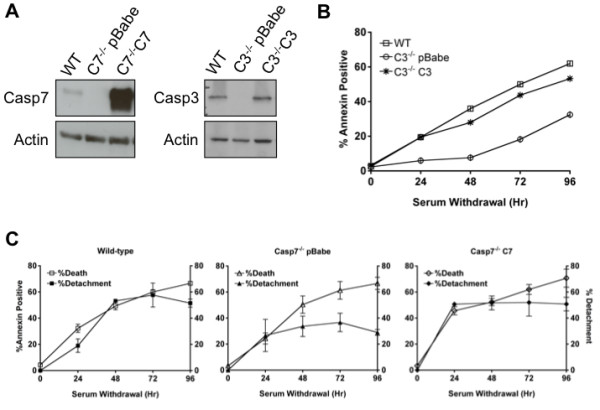
**Reconstitution of caspase-deficient MEFs with caspase-3 or caspase-7 rescues the WT phenotype. (A)** Caspase-deficient MEFs were reconstituted with the appropriate caspase by retroviral transduction and caspase expression was determined by western blot. **(B)** WT, Casp3^-/-^ pBabe and Casp3^-/-^ C3 MEFs were withdrawn from serum for 4 days. At the indicated time points, cell death was determined by Annexin V-FITC staining. Data are presented as the mean ± SEM of at least 3 independent experiments. **(C)** WT, Casp7^-/-^ pBabe and Casp7^-/-^ C7 MEFs were withdrawn from serum for 4 days. At the indicated time points, percent detachment was determined by separating non-adherent from adherent cells and counting with a hemocytometer. Data are presented as the mean ± SEM of at least 3 independent experiments.

Since these MEFs were developed in the absence caspase-3 or caspase-7, we wanted to determine that the cell death and detachment effects were a direct consequence of the absence of the caspase and not due to changes in the development of the MEFs. In order to test these possibilities, we introduced caspase-3 (C3) and caspase-7 (C7) into the caspase-3-deficient MEFs or caspase-7-deficient MEFs, respectively and determined if the reconstituted MEFs revert to a WT phenotype. Caspase-deficient cell lines that stably express C3 or C7 were made by retroviral transduction and selected with puromycin, pBabe-puro was used as an empty vector control. C3 and C7 expression was determined by western blot and C3 expression levels are comparable to endogenous level in WT MEFs, while C7 is expressed at much higher levels (Figure 4A). We found that Casp3^−/−^ MEFs reconstituted with C3 died at the same rate as WT MEFs during a serum withdrawal time course (Figure 4B). Also, in Casp7^−/−^ MEFs reconstituted with C7 the percent of cell death correlated with the percent of cell detachment, suggesting that in the presence of C7 all dead cells detached from the ECM (Figure 4C). Taken together, these data indicate that caspase-3 is the dominant executioner caspase and caspase-7 regulates cell detachment during intrinsic cell death.

## Discussion

While the caspase proteolytic cascade that executes intrinsic apoptosis following cytochrome *c* release is well described, the distinct roles of each caspase during this process are less understood. It has been shown that these caspases have effects on the mitochondria and on upstream events of intrinsic apoptosis, even though they are thought to act downstream of cytochrome *c* release. Caspase-9 has been shown to uncouple the mitochondria and increase ROS production, while cells deficient in caspase-3 or caspase-7 show a delay in the mitochondrial events of intrinsic apoptosis [13,17,20]. Caspase-3 and caspase-7 have been shown to have differential activity toward multiple substrates, including Bid, XIAP and gelsolin, which suggests a non-redundant role for these related caspases [18]. Taken together, these data suggest that caspase-9, caspase-3 and caspase-7 have distinct roles during apoptosis and that there may be a feedback loop on the mitochondria as well as additional upstream functions.

We used IL-3 withdrawal-induced apoptosis and a Casp9DN to study the roles of caspase-9 during intrinsic apoptosis. We found that caspase-9 is necessary to remodel the mitochondria during intrinsic apoptosis and the ability of Casp9DN to inhibit mitochondrial remodeling while having no effect on cytochrome *c* release demonstrates that these events do not have to be linked. Previous studies have demonstrated a role for tBid in the remodeling of mitochondria [10]. However, cleavage of Bid to tBid prior to MOMP does not occur in most forms of intrinsic cell death, including growth factor withdrawal. This is confirmed in this report as Bcl-x_L_ blocks cytochrome *c* release (Data not shown) as well as Bid cleavage (Figure 1) during IL-3 withdrawal. Therefore, if Bid is involved in the mitochondrial remodeling observed during IL-3 withdrawal-induced cell death it would have to be cleaved by a caspase activated after MOMP. Since caspase-8 and caspase-9 cleave caspase-3 at the same site, we reasoned that caspase-9 could cleave Bid at the same site as caspase-8 and result in tBid generation post MOMP [1]. Our data indicate that caspase-9 can cleave Bid at Asp^59^ and suggest that Bid is cleaved in a caspase-9 dependent manner following IL-3 withdrawal of FL5.12 cells. Unfortunately, limitations in the ability to detect endogenously generated tBid prevent us from formally demonstrating this. However, we found that Bid-deficient MEFs display decreased ROS production and that introduction of wild-type, but not cleavage-defective Bid (Bid^D59A^), could restore the ROS production associated with serum withdrawal. Additionally, in the presence of BocD-fmk, ROS increased in the reconstituted cells. Taken together, these data strongly implicate tBid in mitochondrial dysfunction that occurs after MOMP and based on the work of Scorrano and colleagues, tBid is creating a favorable condition for ROS production through mitochondrial remodeling [10].

While caspase-9 cleavage of Bid appears to initiate ROS production following cytochrome *c* release, an effector caspase can extinguish ROS through complete depolarization of the inner mitochondrial membrane. However, the specific effector caspase required for depolarization is not known. Therefore, we used serum withdrawal-induced cell death in Casp KO MEFs to study the distinct effects of caspase-3 and caspase-7 on mitochondrial function during intrinsic apoptosis. Our results show that caspase-3 is decreasing ROS production, while caspase-7 may be required for ROS accumulation. Normally, the mitochondria maintain a membrane potential (ΔΨ_m_) and shuttle electrons across the ETC with minimal ROS production, which can occur at complex III [21]. During apoptosis stimulation, there is a loss of cytochrome *c* from the mitochondria, which is needed to transfer electrons from complex III to IV, resulting in loss of electrons from the ETC and ROS production [22]. If import of substrates to the ETC is stopped by loss of ΔΨ_m_, or electron transport through complex III is blocked, ROS production is diminished [13]. Therefore, after cytochrome *c* release and caspase-9 activation, caspase-3 is needed to inhibit electron transport through the ETC and/or lower ΔΨ_m_ in order to decrease ROS production. This indicates that caspase-9 generation of tBid and remodeling of the mitochondria may represent the ‘point of no return’ in apoptosis and that caspase-3 assures that this does not result in loss of integrity of the apoptotic cell.

Here, we demonstrate that MEFs deficient in caspase-7 die at the same rate as WT MEFs, while caspase-3-deficient MEFs have a delay in cell death during serum withdrawal. The data suggests that caspase-3 is the dominant executioner caspase, while caspase-7 may have other roles, which is in agreement with data on substrate specificity [18]. Consistent with this model, reintroduction of caspase-3 into caspase-3-deficient MEFs resulted in cell lines with expression levels similar to endogenous expression. In contrast, cells could tolerate significantly higher levels of caspase-7 upon reintroduction. Thus it is unlikely that caspase-7 functions primarily to kill cells. We show that caspase-7 functions to detach cells from the ECM, which may suggest that caspase-7 functions to aid in the removal of apoptotic cells. *In vivo*, apoptotic cells can have a profound effect on the microenvironment and it is necessary to regulate these processes and efficiently remove dead cells [19]. Caspase-7 may contribute to this removal process by hastening the detachment of cells from the ECM. Caspases are known to cleave a variety of actin and cytoskeleton components, but the specific components important for detachment and cleaved by caspase-7 are yet to be determined. Interestingly, an early report demonstrated that FAK is an apoptotic substrate that is preferentially cleaved by caspase-7. However, this study was performed in non-adherent cells, therefore it is difficult to fully appreciate the significance of these data [23]. The current studies shed new light on this finding.

## Conclusions

**Figure 5:**
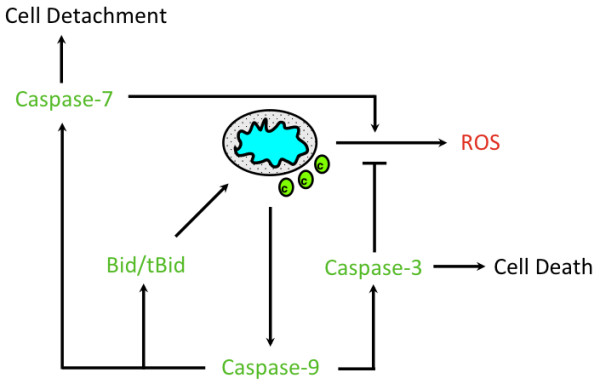
**Model of caspase activation downstream of cytochrome *c *release during apoptosis.** Cell death signals induce MOMP, which leads to cytochrome *c* release and the activation of caspase-9. Caspase-9 can cleave and activate Bid, caspase-7 and caspase-3. tBid can remodel the mitochondria and make conditions favorable for ROS production, which is enhanced by caspase-7 and inhibited by caspase-3.

Taken together, our data suggest distinct roles for caspase-9, caspase-3 and caspase-7 during intrinsic apoptosis (Figure 5). Caspase-9 is activated post cytochrome *c* release and functions to activate effector caspases and Bid in order to remodel the mitochondria. This would assure that cells that have an active apoptosome will still die in the absence of effector caspases. However, cell death is more efficient in the presence of caspase-3, which is the primary executioner of apoptotic death. In contrast, while caspase-7 plays no role in the sensitivity to intrinsic apoptosis, it can cause an accumulation of ROS production and functions to detach cells from the ECM. This is consistent with caspase-7 primarily playing a supportive role in the execution phase of apoptosis. We have found that each caspase has a distinct role in apoptosis, which suggests that the caspases involved in the proteolytic cascade post cytochrome *c* release must work together to effectively and efficiently execute all aspects of apoptosis.

## Methods

### Cell culture

FL5.12 cells are a murine pro-B cell line that is IL-3 dependent and were cultured and transfected as previously described [20,24]. Mouse embryonic fibroblasts (MEFs) were cultured in Dulbecco’s Modification of Eagle’s Medium (Cellgro) supplemented with 10% fetal bovine serum (FBS, Cellgro), 1% non-essential amino acids (Cellgro), 1 mM sodium pyruvate (Cellgro), 55 μM 2-Mercaptoethanol (Gibco), 2 mM L-glutamate (Cellgro) and 100 U/ml Penicillin-Streptomycin (Cellgro) at 37°C in a humid 5% CO_2_ incubator. When indicated, BocD-fmk was used at a concentration of 100 μM. MEFs were infected with retrovirus generated by transfecting the ΦNX-Ecotropic cell line (Nolan lab, Stanford University) with a plasmid (pBabe-puro, pBabe-Bid or pBabe-Bid^D59A^) using Lipofectamine (Invitrogen) [25].

ΦNX-Ecotropic packaging cell lines (Nolan lab, Stanford University) were transfected with pBabe-puro, Casp3 pBabe-puro or Casp7 pBabe-puro using Lipofectamine (Invitrogen). Target MEFs were seeded in 6-well plates and allowed to grow for 24 hours and then infected with viral supernatants at 24, 28, and 32 hours using Polybrene Infection / Transfection Reagent (Millipore). After 24 hours viral supernatants were removed from the target cells and replaced with fresh medium for 24-72 hours and then they were selected with 2.5 μg/ml puromycin (Sigma).

### Cell death induction and analysis

IL-3 withdrawal-induced cell death in FL5.12 cells was conducted as previously described [13]. For serum withdrawal-induced apoptosis, medium was aspirated from MEFs, they were washed with PBS and serum-free medium was added for indicated time points. Cell death was assayed by Annexin V-FITC and propidium iodide and analyzed on a FACSCanto flow cytometer (BD Biosciences). Percent detachment was determined by separating non-adherent and adherent cells and counting on a hemocytometer. Significance was determined by t-test using Prism software.

### Microscopy and mitochondrial assays

Fluorescent-confocal microscopy for actin and DNA was conducted by fixing cells as previously described and staining with phalloidin (Cell Signaling) for 20 min and mounting the coverslips with Prolong Gold with DAPI (Molecular Probes) [13]. Mitochondrial membrane potential and ROS production were assayed as previously described [13,20].

### Caspase-9 cleavage of bid

Bid mutants (Bid^D98A^, Bid^D75A^, and Bid^D59A^) were created by site directed mutagenesis. *In vitro* translated Bid, Bid^D98A^, Bid^D75A^, or Bid^D59A^ was exposed to 0, 5, 10 Units of recombinant caspase-9 for 90 min at 37°C and Bid cleavage was assessed by western blot.

### Western blotting

Western blotting was performed as previously described [24]. Primary antibodies: mouse anti-Bid (Stanley Korsmeyer), rabbit anti-caspase-3 (Cell Signaling), rabbit anti-caspase-7 (Cell Signaling), and rabbit anti-Actin (Sigma). Secondary antibodies: horseradish peroxidase-conjugated sheep anti-mouse and horseradish peroxidase-conjugated donkey anti-rabbit (Amersham). Proteins were detected by chemiluminescence (Amersham).

## Competing interests

The authors declare that they have no competing interests.

## Authors’ contributions

MB designed and performed experiments and prepared the manuscript. LRM designed and performed experiments. RLDG performed experiments. EC designed and performed experiments. LHB oversaw project, designed experiments and prepared the manuscript. All authors read and approved the final manuscript.
